# Contemporary epidemiological landscape of pediatric non-Hodgkin lymphoma: a global burden of disease analysis

**DOI:** 10.3389/fonc.2025.1606627

**Published:** 2025-06-09

**Authors:** Xiaobo Luo, Pin Xie, Min Zhou, Yi Xia, Yu Gao, Hui Li

**Affiliations:** ^1^ Chengdu Women’s and Children’s Central Hospital, School of Medicine, University of Electronic Science and Technology of China, Chengdu, China; ^2^ Department of Orthopedics, Chengdu Shuangliu Hospital of Traditional Chinese Medicine, Chengdu, China

**Keywords:** non-Hodgkin lymphoma, global disease burden, prevalence, temporal trend, health inequality

## Abstract

**Introduction:**

This study evaluated the distribution characteristics, influencing factors, and future trends of non-Hodgkin lymphoma (NHL) burden in children and adolescents globally from 1990 to 2021.

**Methods:**

Data were obtained from the Global Burden of Disease Study database. Multiple analytical methods were used, including Joinpoint regression, age-period-cohort analysis, decomposition analysis, frontier analysis, health equity analysis, and Bayesian age-period-cohort (BAPC) model.

**Results:**

In 2021, the global age-standardized prevalence rate was 3.177/100,000, with a disability adjusted life year (DALY) rate of 13.535/100,000. The prevalence demonstrated a fluctuating downward trend during 1990-2021. Age, period, and cohort effects significantly influenced disease patterns. While population growth drove prevalence increase, population aging and epidemiological factors had negative impacts. Disease burden showed a non-linear negative correlation with Socio-demographic Index (SDI). Over the past nearly 30 years, health inequality has intensified, as some African regions have shown relatively low prevalence rates due to limited resource Settings, which have restricted disease diagnosis and reporting, compared with the developed areas with high prevalence. The BAPC model predicted further decrease from 2022-2036.

**Discussion:**

Despite overall decline, significant regional differences and health inequalities persist, suggesting future focus on targeted prevention, optimized resource allocation, and improved treatment.

## Introduction

Lymphoma is a malignant tumor that originates in the lymph nodes and lymphoid tissues (1). Its development is associated with the malignant transformation of immune cells, which results from the proliferation and differentiation of lymphocytes. Based on histopathological changes, lymphoma is classified into two major types: Hodgkin lymphoma (HL) and non-Hodgkin lymphoma (NHL), accounted for about 40% and 45% of lymphomas, respectively (2). Among these, NHL is particularly notable, ranking as the fourth most common malignancy in children and showing an even higher incidence in adolescents (3). As one of the most common cancers in children and adolescents, NHL poses a serious threat to the health and quality of life of this population (4).

Factors influencing the risk of developing NHL include immune diseases, medications, infections, lifestyle, genetics, ethnicity, family history, and occupational factors. For example, obesity has been identified as a risk factor for diffuse large B-cell lymphoma (DLBCL). Approximately 85-90% of NHL cases originate from B cells, while the remaining cases arise from T cells or NK cells (5).In recent years, with the rapid development of medical technology and the continuous improvement of living standards, the survival rate of children and adolescents with NHL has significantly increased (6).The current overall survival rate exceeds 80% (3). However, the incidence and mortality of NHL in children and adolescents still show an increasing trend globally, and there are significant regional differences in the distribution of disease burden (6). In addition, NHL survivors often need to receive long-term follow-up and rehabilitation treatment (7, 8), facing serious complications and psychosocial problems. Therefore, NHL has become one of the major public health problems threatening the health of children and adolescents, bringing heavy economic burden and psychological pressure to patients’ families and society.

A comprehensive understanding of the epidemiological characteristics and influencing factors of NHL in children and adolescents is crucial for early prevention, standardized diagnosis and treatment, and rational use of drugs for NHL. However, current research on the global disease burden of NHL in children and adolescents is still relatively limited, and there is a lack of systematic analysis with long time spans and multi-regional comparisons. Most of the existing studies are limited to a single country or region (4, 8), and the data sources and research methods used are not consistent, making it difficult to conduct an overall assessment of the global prevalence of NHL in children and adolescence (4, 8).

Since its launch in 1990, the Global Burden of Disease (GBD) study has systematically evaluated the incidence, mortality, and disability rates of hundreds of diseases and injuries in different populations by integrating epidemiological data from various countries and regions (9). GBD has provided an important data foundation and methodological reference for comprehensively understanding disease trends and formulating health policies (10). In the field of NHL, GBD provides key indicators such as incidence, prevalence, mortality, and disability-adjusted life years in various regions and age groups, providing a valuable data resource for conducting NHL global disease burden research (11).

Based on the above background, this study intends to use the GBD database to systematically evaluate the epidemiological characteristics, spatial-temporal distribution characteristics and influencing factors of NHL in children and adolescents worldwide from 1990 to 2021, and to predict future prevalence trends. The study will apply a variety of statistical methods to quantitatively assess the epidemiological characteristics, time trends, influencing factors, and regional differences of NHL in children and adolescents, and use spatial analysis techniques to intuitively display the spatial-temporal evolution process of the global NHL disease burden in children and adolescents. The research results will help to clarify the priorities and difficulties of current NHL prevention and treatment work, and provide a reference for the formulation of NHL prevention and control plans and health resource allocation schemes worldwide. At the same time, this study will also provide methodological references for the spatial-temporal analysis of other chronic disease burdens.

## Materials and methods

### Data sources and indicator calculation

The data used in this study were obtained from the GBD study. Epidemiological data on NHL in children and adolescents from 204 countries and regions worldwide from 1990 to 2021 were downloaded from the GBD website, including indicators such as crude prevalence rate, age-standardized prevalence rate (ASPR), and disability-adjusted life years (DALYs). Corresponding demographic data were also downloaded for the standardized calculation of prevalence rates. The main calculated indicators included: prevalence rate, ASPR, and age-standardized DALY rate (ASDR).

### Statistical analysis methods

Descriptive analysis of disease burden: Descriptive analysis was performed on the prevalence rate and DALYs of NHL in children and adolescents (0-19 years) globally and in 21 regions from 1990 to 2021. The number of incident cases, crude prevalence rate, ASPR, number of DALYs, ASDR, and the percentage change of these indicators from 1990 to 2021 were calculated for each region and year. Disease burden heatmaps were plotted to show the distribution of NHL in children and adolescents worldwide.

Joinpoint regression analysis: The Joinpoint regression model was applied to analyze the trends in ASPRs of NHL in children and adolescents from 1990 to 2021. The standard errors of ASPRs were calculated using R software, and the data were imported into the Joinpoint software. Time was set as the independent variable, age-standardized rate as the dependent variable, region as the grouping variable, and a maximum of 5 joinpoints were allowed with a 95% confidence interval for parameter testing. The results were expressed as annual percent change (APC).

Age-period-cohort analysis: To explore the temporal trends of NHL in children and adolescents, data from 1992 to 2021 were divided into 5-year periods after excluding data from 1990-1991, and uploaded to the APC website for age, period, and cohort effect analysis. The influence of each effect on the prevalence of NHL was calculated, and graphs of relative risk changes with age, period, and cohort were plotted. The local drifts percentages of prevalence risk in each age group compared to the previous age group were calculated.

Decomposition analysis: To comprehensively assess the driving forces behind changes in the prevalence rate NHL from 1990 to 2021, we applied Das Gupta’s decomposition method. This approach allowed us to quantify the contributions of three major components: population growth, changes in population age structure, and epidemiological changes. The analysis was stratified by global level, sex, and Socio-demographic Index (SDI) regions.

Epidemiological changes were defined as alterations in age-specific and population-standardized prevalence rates over time, after adjusting for demographic changes. Specifically, they reflect shifts in disease risk that are not attributable to population size or age distribution alone—such as improvements in diagnosis, treatment, or exposure to risk factors. For each year y, the total number of cases was calculated using the formula (12):


$${\text{Prevalance}}_{ay,py,ey}=\sum_{i=1}^{20}(a_{i,y}\times p_{y}\times e_{i,y})$$

where $a_{i,y}$ is the proportion of the population in age group i, $p_{y}$ is the total population in year *y*, and $e_{i,y}$ is the age-specific prevalence rate. The decomposition isolates the effect of each factor by varying one component at a time while keeping the others constant, thereby minimizing the interaction effects among components.

While this method assumes that the three components operate independently, potential interactions (e.g., age structure affecting epidemiological rates) are partially addressed through sequential standardization. Nevertheless, it is important to interpret results in the context of these assumptions.

Frontier analysis: The data envelopment analysis (DEA) method was used to evaluate the relationship between the disease burden of NHL and the SDI. The free disposal hull (FDH) model was used to fit the nonlinear production frontier, and the locally weighted regression scatter plot smoothing (LOWESS) method was used to generate a smoothed frontier. Super efficiency points (points below the frontier boundary) were removed to avoid the influence of outliers.

Health inequality analysis: The slope index of inequality (SII) and concentration index (CI) of the ASPR of NHL were calculated. SII reflects the absolute inequality of NHL, while CI reflects the relative inequality. The SII and CI were calculated for 1990 and 2021, and scatter plots of SII and CI were drawn to compare the changes in health inequalities between the two time points.

Prevalence prediction analysis: The Bayesian Age-Period-Cohort (BAPC) model was used to predict the ASPR of NHL from 2022 to 2036. The BAPC model considers the age, period, and cohort effects, sets prior distributions, infers the three effects a posteriori by combining the sample data, and finally combines the three effects to predict the prevalence rate.

All data analyses and plotting were performed using R 4.2.0, and the BAPC model was implemented using the BAPC package. Statistical tests were considered statistically significant at P<0.05.

## Results

### Temporal trends in the burden of NHL

In 2021, the global number of incident cases of NHL in children and adolescents was 227,438.038 (95% uncertainty interval [UI]: 189,379.563-276,291.764), and the ASPR was 3.177/100,000 (95% UI: 2.634-3.874). In terms of prevalence number, among the 21 regions, South Asia had the highest number of incident cases in 2021, with 46,144.590 (95% UI: 34,410.725-62,856.516), while Oceania had the lowest number of incident cases in 2021, with 617.558 (95% UI: 346.587-1,046.539). In terms of ASPR, Western Europe had the highest ASPR, reaching 7.494/100,000 (95% UI: 6.275-8.985), while Central Sub-Saharan Africa had the lowest ASPR, at 1.233/100,000 (95%UI:0.721-1.832). The region with the largest annual percentage change increase in ASPR of NHL from 1990 to 2021 was Southern Sub-Saharan Africa, with an increase of 85.469%, while the region with the largest decrease was Central Sub-Saharan Africa, with a decrease of 38.817% (**Table 1**).

**Table 1:** Number of incident cases, age-standardized prevalence rate, and percentage change from 1990 to 2021 of NHL in children and adolescents globally and in 21 GBD regions in 2021.

Location	Prevalence Number (95%UI)	ASPR (95%UI) per 100,000	ASPR Percentage change (1990 to 2021)
Global	227438.038 (189379.563,276291.764)	3.177 (2.634,3.874)	-6.377
Andean Latin America	3047.762 (2051,4546.606)	4.678 (3.147,6.979)	45.109
Australasia	983.595 (690.058,1355.93)	4.692 (3.293,6.467)	-19.614
Caribbean	1866.483 (1280.626,2581.359)	4.533 (3.085,6.295)	-18.794
Central Asia	3114.155 (2389.212,4068.584)	3.299 (2.539,4.299)	-13.579
Central Europe	2566.375 (2128.52,3108.254)	3.925 (3.25,4.762)	15.175
Central Latin America	6517.488 (5338.168,7964.042)	2.737 (2.232,3.357)	6.316
Central Sub-Saharan Africa	2461.435 (1428.664,3659.245)	1.233 (0.721,1.832)	-38.817
East Asia	23943.202 (18666.551,31087.109)	2.523 (1.959,3.285)	-18.05
Eastern Europe	5267.548 (4616.8,6001.257)	4.152 (3.631,4.743)	-36.598
Eastern Sub-Saharan Africa	27117.221 (17480.091,39992.153)	4.352 (2.816,6.399)	-23.166
High-income Asia PaUIfic	2999.294 (2207.526,4075.111)	3.479 (2.543,4.755)	14.506
High-income North America	9839.157 (8631.957,11154.826)	3.89 (3.401,4.423)	-33.303
North Africa and Middle East	22488.672 (17848.67,28962.066)	3.484 (2.764,4.488)	11.428
Oceania	617.558 (346.587,1046.539)	3.515 (1.99,5.929)	49.866
South Asia	46144.59 (34410.725,62856.516)	2.509 (1.857,3.442)	7.411
Southeast Asia	13716.243 (10777.581,18401.369)	2.171 (1.699,2.92)	12.473
Southern Latin America	1581.18 (1173.784,2132.932)	2.879 (2.136,3.884)	2.026
Southern Sub-Saharan Africa	2678.056 (1937.984,3573.641)	3.145 (2.271,4.202)	85.469
Tropical Latin America	3965.321 (3270.58,4723.158)	2.155 (1.77,2.574)	-17.252
Western Europe	19145.864 (16067.057,22909.469)	7.494 (6.275,8.985)	0.605
Western Sub-Saharan Africa	27376.839 (15806.399,39810.32)	3.618 (2.115,5.232)	-12.721

The DALYs caused by NHL were 962,473.898 (95% UI: 769,887.139-1,207,937.694), and the ASDR was 13.535/100,000 (95% UI: 10.772-17.052). Among the other 21 regions, South Asia had the highest DALYs caused by NHL in 2021, at 229,744.212 (95% UI: 175,628.271-310,401.205), while Australasia had the lowest DALYs caused by NHL in 2021, at 727.547 (95% UI: 582.512-896.601). In terms of ASDR, the Eastern Sub-Saharan Africa region had the highest ASDR, at 33.526/100,000 (95% UI: 23.028-48.271), while High-income North America had the lowest ASDR, at 3.039/100,000 (95% UI: 2.819-3.267). The Southern Sub-Saharan Africa region had the largest annual percentage change increase in ASDR from 1990 to 2021, at 55.546%, while the East Asia region had the largest decrease, at 70.117% (**Table 2**).

**Table 2:** Number of DALYs, age-standardized DALY rate, and percentage change in DALYs from 1990 to 2021 of NHL in children and adolescents globally and in 21 GBD regions in 2021.

Location	DALYs Number (95%UI)	ASDR (95%UI) per 100,000	ASDR Percentage change (1990 to 2021)
Global	962473.898 (769887.139,1207937.694)	13.535 (10.772,17.052)	-36.448
Andean Latin America	8583.493 (6450.078,11381.85)	13.251 (9.948,17.586)	-44.883
Australasia	727.547 (582.512,896.601)	3.463 (2.771,4.27)	-62.464
Caribbean	9711.261 (5785.524,14540.3)	23.667 (13.922,35.575)	-33.165
Central Asia	10186.061 (8170.081,12713.023)	10.81 (8.696,13.457)	-43.14
Central Europe	4063.362 (3622.042,4537.429)	6.211 (5.52,6.957)	-52.678
Central Latin America	20183.721 (17235.251,23720.542)	8.461 (7.188,9.997)	-46.491
Central Sub-Saharan Africa	21245.804 (12373.178,31433.753)	10.618 (6.236,15.701)	-49.354
East Asia	61026.175 (48003.027,77841.809)	6.453 (5.056,8.249)	-70.117
Eastern Europe	8332.037 (7517.902,9294.645)	6.55 (5.898,7.322)	-64.48
Eastern Sub-Saharan Africa	208655.307 (142823.427,301291.464)	33.526 (23.028,48.271)	-40.467
High-income Asia PaUIfic	4105.831 (3714.383,4550.926)	4.659 (4.21,5.167)	-59.358
High-income North America	7815.177 (7273.484,8379.577)	3.039 (2.819,3.267)	-60.646
North Africa and Middle East	57192.284 (46558.395,74437.361)	8.877 (7.221,11.548)	-50.463
Oceania	1753.272 (1028.978,2780.756)	10.04 (5.921,15.892)	20.804
South Asia	229744.212 (175628.271,310401.205)	12.488 (9.481,16.999)	-37.773
Southeast Asia	62340.029 (49920.039,82727.02)	9.855 (7.851,13.133)	-37.633
Southern Latin America	4071.416 (3320.374,4940.616)	7.439 (6.057,9.041)	-45.015
Southern Sub-Saharan Africa	14885.533 (10380.406,20100.058)	17.494 (12.168,23.656)	55.546
Tropical Latin America	13912.833 (11811.395,16011.537)	7.554 (6.382,8.72)	-54.269
Western Europe	11781.397 (10701.146,12987.237)	4.569 (4.141,5.045)	-51.12
Western Sub-Saharan Africa	202157.147 (127620.288,276599.393)	26.72 (17.056,36.403)	-31.147

### Joinpoint regression analysis

**Figure 1:**
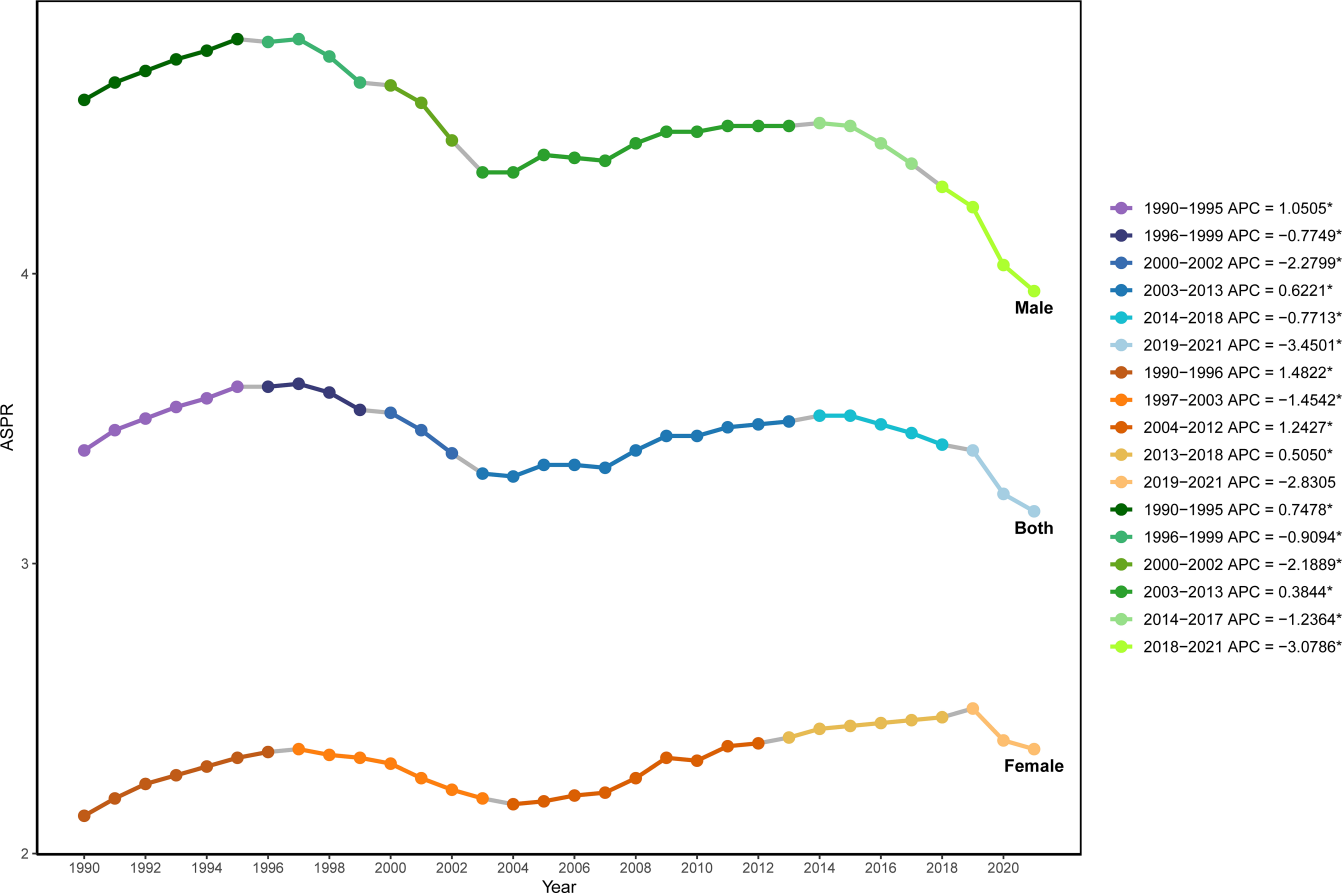
Joinpoint regression analysis of the age-standardized prevalence rate of NHL in children and adolescents globally from 1990 to 2021.

The results of Joinpoint regression analysis showed that during 1990 to 2021, the global ASPR of NHL exhibited an overall fluctuating downward trend, with five significant turning points detected in 1995, 1999, 2002, 2013, and 2019 (**Figure 1**). Specifically, from 1990 to 1995, the global ASPR of NHL showed an upward trend, with an annual percent change (APC) of 1.05% (P<0.05). From 1996 to 1999, the prevalence rate turned to decline, with an APC of -0.77% (P<0.05). From 2000 to 2002, the prevalence rate continued to decline, and the magnitude of decline further increased compared to the previous period, with an APC of -2.28% (P<0.05). From 2003 to 2013, the prevalence rate showed an upward trend again, with an APC of 0.62% (P<0.05). From 2014 to 2018 and from 2019 to 2021, the prevalence rate turned to a downward trend again, with the rate of decline gradually accelerating, and the APCs were -0.77% (P<0.05) and -3.45% (P<0.05), respectively.

Further analysis revealed that the trends in the standardized prevalence rate of NHL in males from 1990 to 2021 were similar to those in the overall population, showing a fluctuating downward trend, while the prevalence rate in females showed an upward trend. For males, the APCs were 0.75% P<0.05), -0.91% (P<0.05), -2.19% (P<0.05), 0.38% (P<0.05), -1.24% (P<0.05), and -3.08% (P<0.05) for the periods of 1990-1994, 1995-1999, 2000-2002, 2003-2013, 2014-2017, and 2018-2021, respectively. For females, the APCs were 1.48% (P<0.05), -1.45% (P<0.05), 1.24% (P<0.05), 0.51% (P<0.05), -2.83% (P<0.05) for the periods of 1990-1996, 1997-2003, 2004-2011, 2012-2018, and 2019-2021, respectively.

### Age-period-cohort effect analysis of NHL prevalence

**Figure 2:**
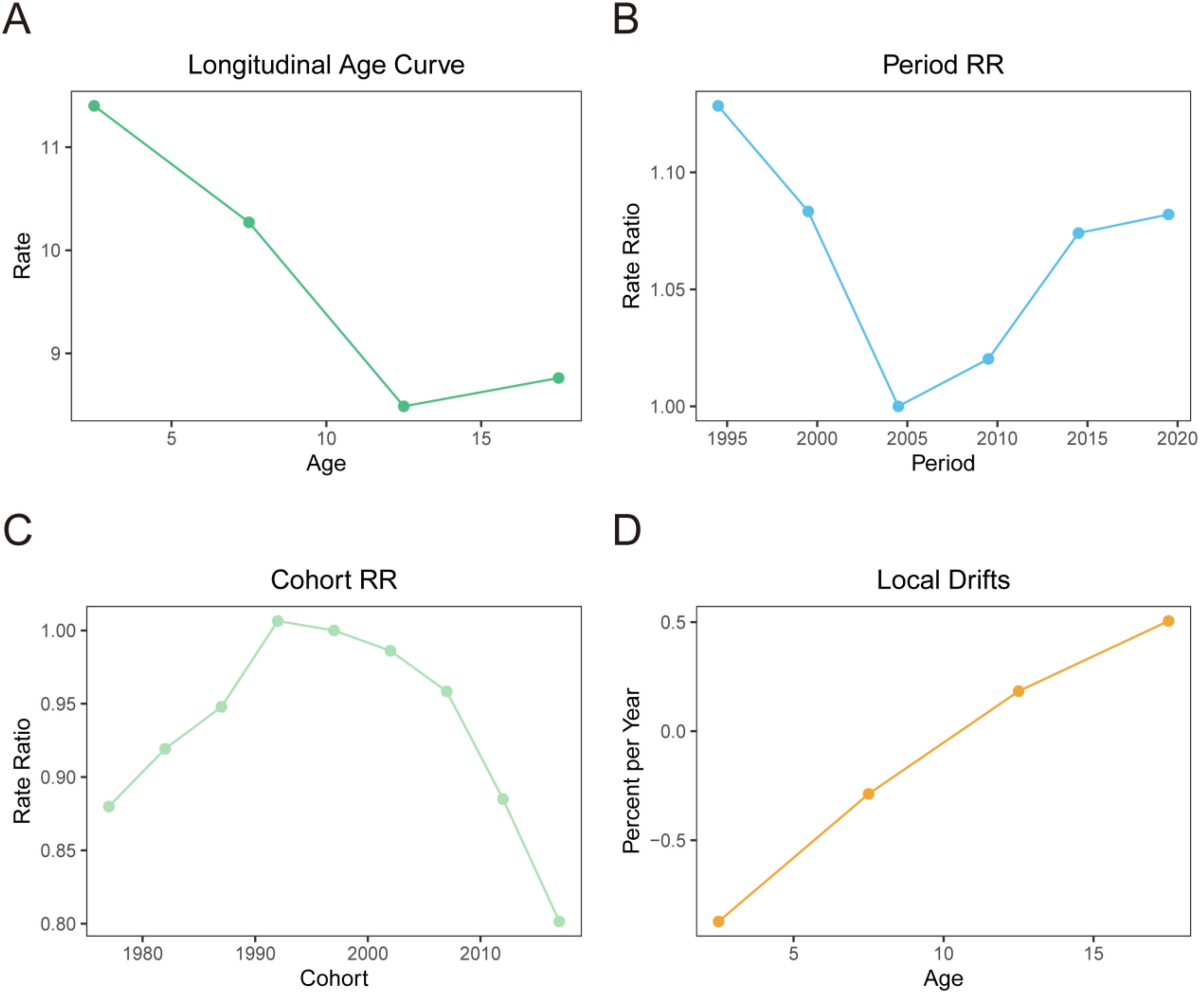
Age-period-cohort effect analysis of the prevalence of NHL in children and adolescents globally from 1990 to 2021. **(A)** Results of the age effect analysis. The horizontal axis represents age, and the vertical axis represents relative risk. **(B)** Results of the period effect analysis. The horizontal axis represents period, and the vertical axis represents relative risk, with 2004 as the reference period. **(C)** Results of the cohort effect analysis. The horizontal axis represents birth cohort, and the vertical axis represents relative risk, with the 1992-1997 cohort as the reference cohort. **(D)** Local drift percentages of prevalence risk of NHL in children and adolescents in different age groups. The horizontal axis represents age group, and the vertical axis represents local drift percentage, which represents the percentage change in prevalence risk of each age group compared to the previous age group.

There were significant age effects, period effects, and cohort effects on the prevalence risk of NHL. In terms of age effects, the prevalence risk of NHL gradually decreased with increasing age, reaching the lowest level at 12.5 years old (Rate=8.486/100,000) and slightly increasing to 8.761/100,000 at 17.5 years old (**Figure 2A**). This suggests that younger children may be more susceptible, with 12.5 years old being a critical turning point for prevalence risk, and there may be a slight rebound in late adolescence (17.5 years old).

The period effect analysis found that the prevalence risk of NHL showed an overall decreasing and then increasing trend from 1994 to 2019. Specifically, it showed a rapid decline from 1994 to 2004, followed by a slow increase after 2004, but the prevalence risk in 2019 was still lower than that in 1994 (**Figure 2B**). Taking 2004 as the reference, the relative risk (Rate Ratio, RR) of NHL in 1994 was 1.128, and it decreased to 1.082 by 2019. This indicates that the impact of period effects on the risk of NHL showed an overall weakening trend, and although there was a slight increase in recent years, it did not exceed the historical high point.

The cohort effect analysis results showed that there were significant differences in the prevalence risk of NHL among children an d adolescents in different birth cohorts, indicating a clear cohort effect (**Figure 2C**). Taking the 1992-1997 birth cohort as the reference (RR=1), the RR of the 1972-1977, 1977-1982, 1982-1987, and 1987-1992 birth cohorts increased from 0.880 to 1.00. After 1997, the RR continued to decrease, reaching 0.802 in the 2012-2017 birth cohort. This suggests that there are generational differences in the decline of prevalence risk of NHL born in different eras.

Taking the birth cohort of 1992-1997 as the reference (RR = 1), the RR of the birth cohorts of 1972-1977, 1977-1982, 1982-1987, 1987-1992, and 1992-1997 increased from 0.880 to 1.00. After 1997, the RR continued to decline, and by the birth cohort of 2012-2017, the RR decreased to 0.802. This suggests that there are intergenerational differences in the decline of the risk of NHL among children and adolescents in different birth years, and such differences may be related to generational characteristics such as improvements in lifestyle and changes in environmental factors.

Further analysis of the local drift percentages of different age groups, which refers to the percentage change in disease risk for each age group compared to the previous age group. The results showed significant differences in the local drift percentage between different age groups. The local drift percentage in the age group of 2.5 to 7.5 years was negative, indicating a declining trend in the incidence of NHL. This decline gradually slowed with increasing age. However, after 12.5 years, the local drift percentage became positive and continued to rise, suggesting a rebound in disease risk after puberty (**Figure 2D**).

### Analysis of global prevalence trends in NHL among children aged 12–23 months

**Figure 3:**
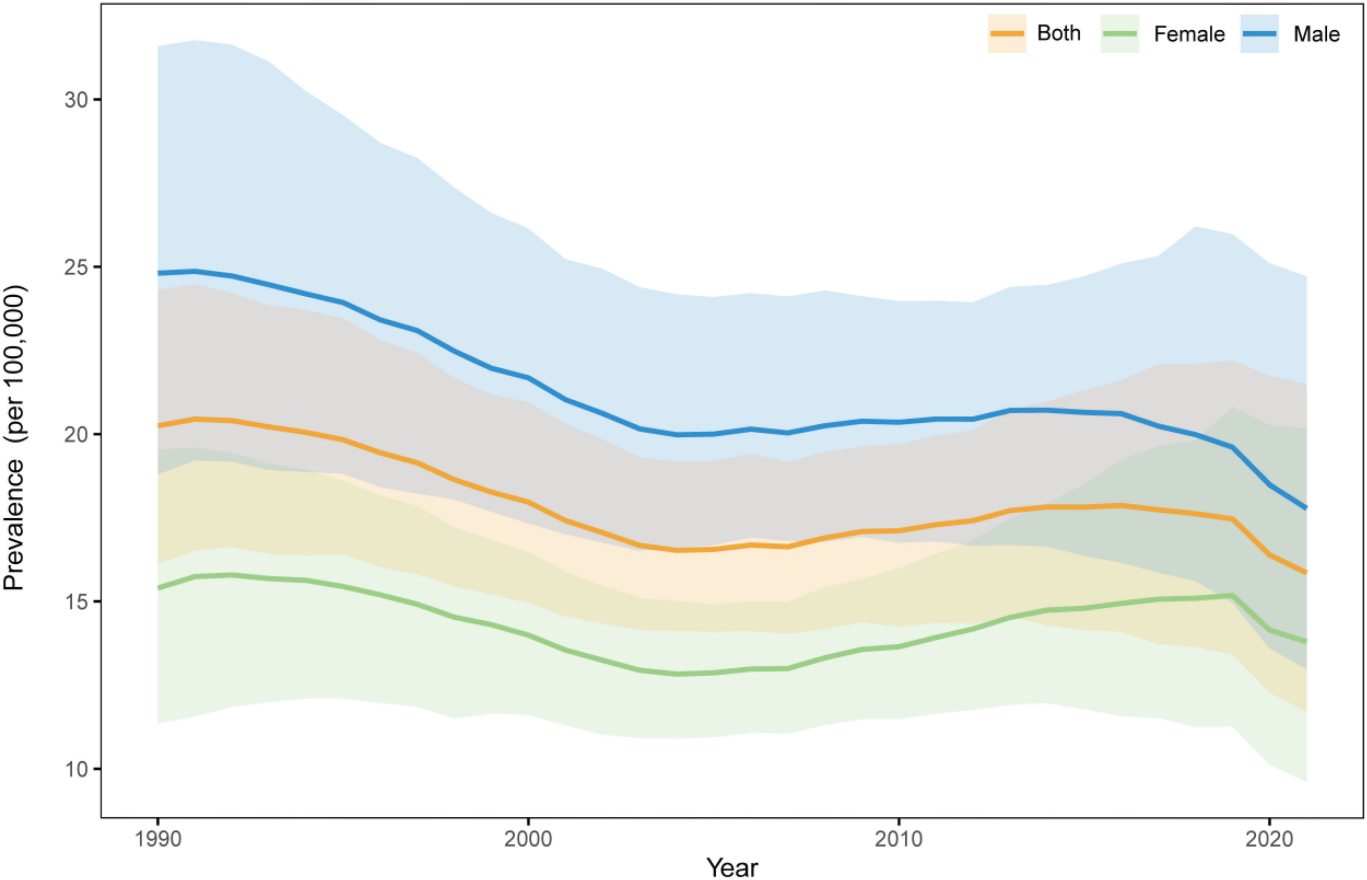
Analysis of the global incidence of NHL in children aged 12-23 months from 1990 to 2021.

To investigate the epidemiological characteristics of NHL among children under five years old from 1990 to 2021, we conducted an in-depth analysis using data for the 12-23 months age group, which was the only available age group in the GBD database. The results demonstrated a fluctuating downward trend in NHL prevalence among children in this age group during the study period (**Figure 3**), decreasing from 20.25/100,000 in 1990 to 15.86/100,000 in 2021, representing a reduction of 21.68%.

Gender-stratified analysis revealed that while both boys and girls followed the overall declining trend, significant gender disparities persisted. Specifically, the prevalence among boys decreased from 24.81/100,000 in 1990 to 17.79/100,000 in 2021, showing a reduction of 28.30%. For girls, the prevalence declined from 15.40/100,000 to 13.80/100,000, with a reduction of 10.37%. Notably, throughout the entire study period, the prevalence among boys consistently remained significantly higher than both the overall average and the prevalence among girls, and although the gender gap narrowed, it continued to persist.

### Decomposition analysis of changes in the prevalence rate of NHL

**Figure 4:**
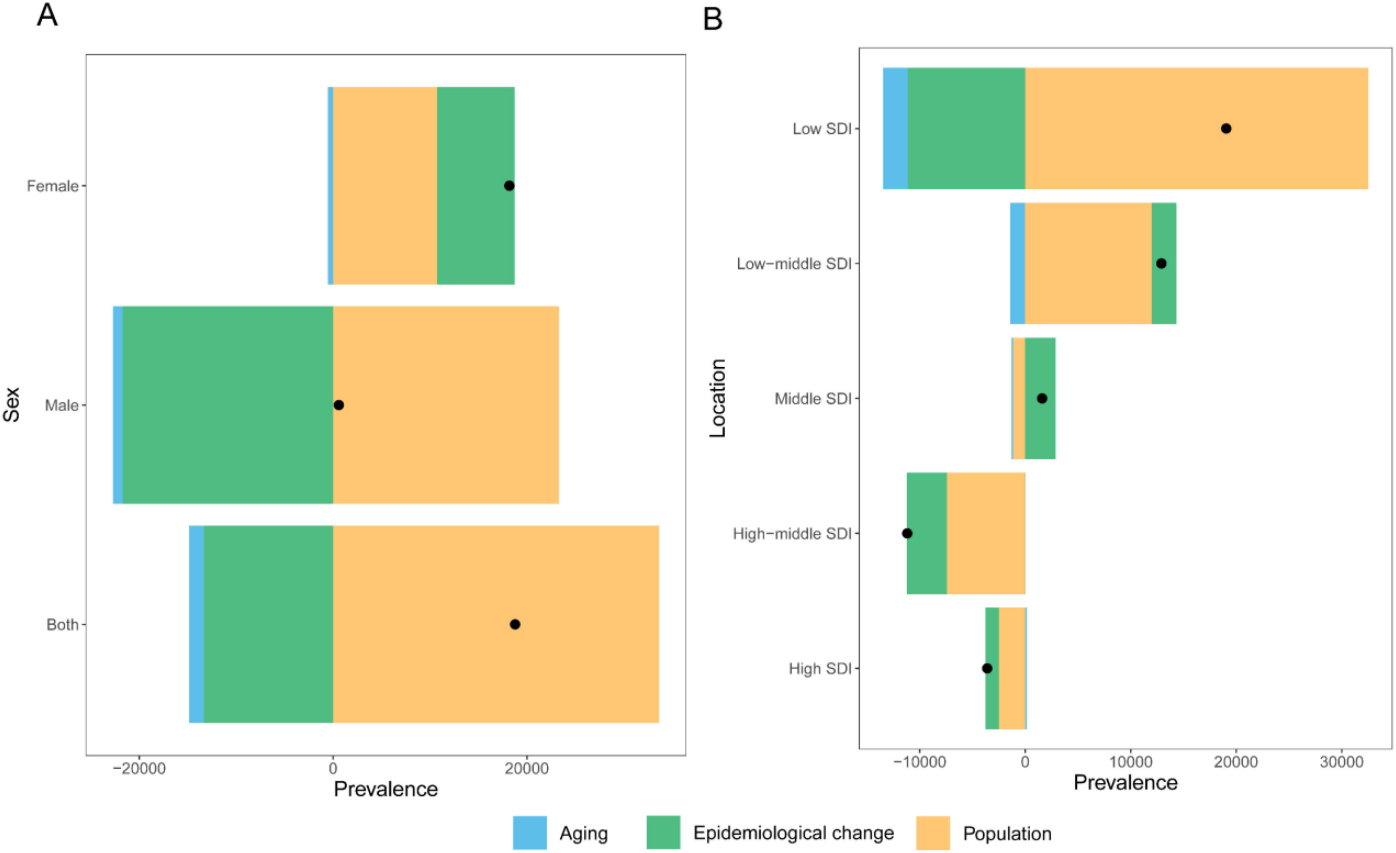
Decomposition analysis of changes in the disease prevalence rate burden of NHL in children and adolescents from 1990 to 2021. **(A)** Decomposition analysis of prevalence across the globe and between different genders. **(B)** Decomposition analysis of prevalence in different SDI regions.

Globally, population growth was the primary factor driving the increase in the prevalence rate of NHL, contributing 179.03% of the prevalence rate change. In contrast, population aging (-8.03%) and epidemiological factors (-71.00%) had negative effects on the increase in prevalence rate (**Figure 4A**). This indicates that although population aging and disease prevention factors have reduced the prevalence risk to a certain extent, the expansion of population size is still the main reason for the increase in the number of incident cases.

By gender, the prevalence rate of NHL in male children and adolescents increased slightly, with the contribution of population growth as high as 3936.56%, while population aging (-171.50%) and epidemiological factors (-3665.05%) largely offset some of the growth. The trend of prevalence rate change in females was similar to that in the overall population, with the contributions of population growth, population aging, and epidemiological factors being 58.97%, -2.89%, and 43.92%, respectively (**Figure 4A**).

Further analysis by SDI showed that as the SDI level increased, the prevalence rate of NHL showed a downward trend, and the contribution of population aging to prevalence rate changes also varied (**Figure 4B**). Among them, the contribution of population aging was lowest in low SDI regions (-12.24%), followed by low-middle SDI regions (-11.08%). In low SDI and low-middle SDI regions, population factors were the most important factors driving the increase in prevalence rate, with contribution rates as high as 170.67% and 92.80%, respectively. In middle SDI regions, population factors had a major negative effect on the increase in prevalence rate (-71.53%), and in this region, the contribution of epidemiological factors to prevalence rate reached 180.6%, suggesting that changes in population structure in this region actually favored a decrease in disease prevalence, but possibly due to significant influences of epidemiological factors such as improved disease diagnostic capabilities, improved surveillance systems, changes in environmental factors, and changes in lifestyle, the prevalence rate ultimately increased. In high SDI and high-middle SDI regions, population factors were the most important factors for the decrease in prevalence rate, and the contribution increased with increasing SDI, which may be due to the continued decline in birth rates in high SDI regions and a more significant decrease in the population base of children and adolescents.

### Relationship between the burden of NHL and socio-demographic development level

**Figure 5:**
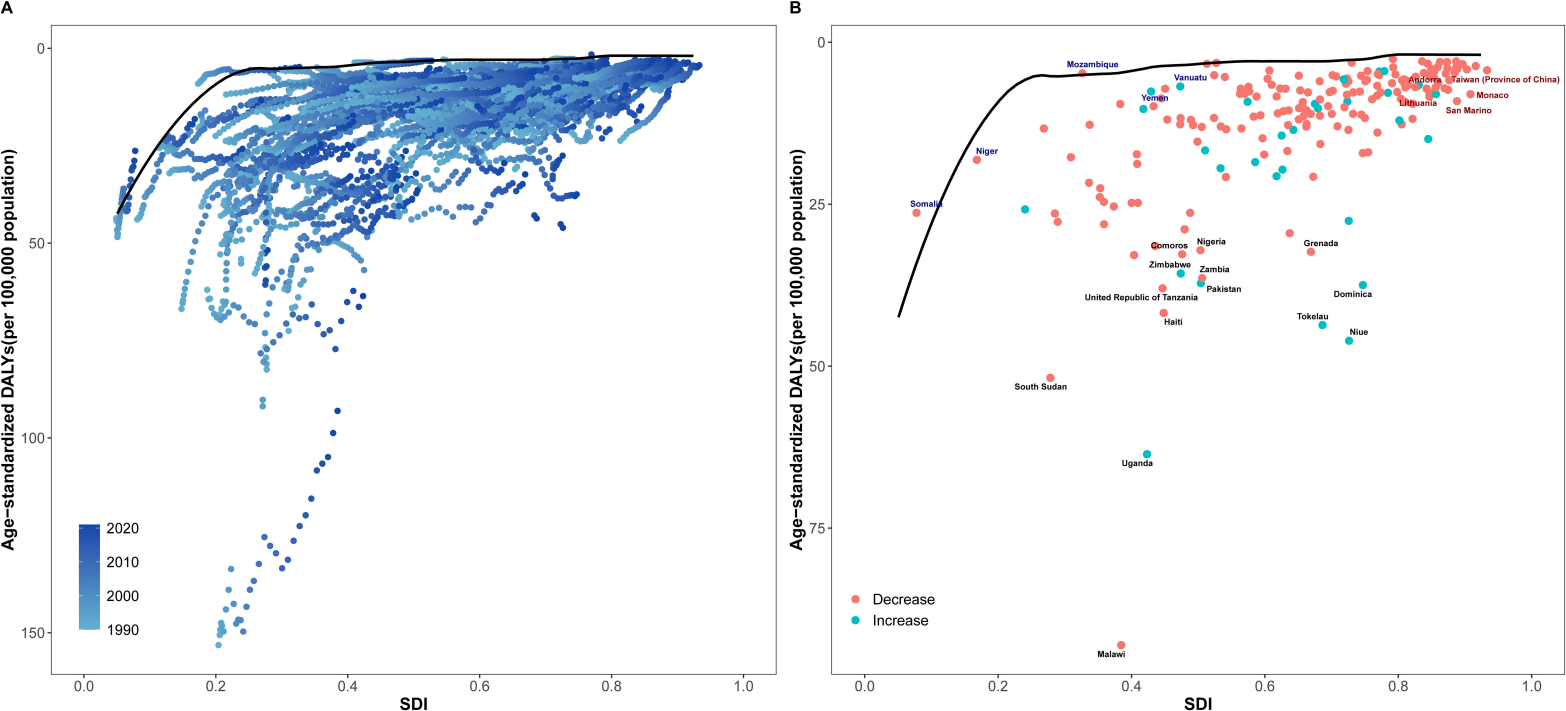
Relationship between SDI and age-standardized DALY rate of NHL in children and adolescents from 1990 to 2021. **(A)** Scatter plot of SDI and age-standardized DALY rate of NHL in children and adolescents from 204 countries from 1990 to 2021. The black curve is the frontier production function fitting curve, indicating the minimum DALY rate at a given SDI level. Points of different colors represent different years. **(B)** Trends in age-standardized DALY rates of NHL in children and adolescents in some countries in 2021. The black solid line is the frontier production function fitting curve, and the colored dotted lines represent the changes in DALY rates of each country. Black fonts represent the 15 points with the largest distance differences; blue fonts represent the 5 countries with the smallest distance differences among low SDI countries; red fonts represent the 5 countries with the largest distance differences among high SDI countries.

The results of DEA showed that the DALY rate of NHL was non-linearly negatively correlated with SDI. As SDI increased, the DALY rate of NHL showed an overall downward trend, but the rate of decline gradually slowed down (**Figure 5A**). Specifically, when SDI was low (<0.2), the DALY rate declined relatively rapidly; after SDI exceeded 0.2, the rate of decline in DALY rate slowed down; when SDI reached above 0.4, the DALY rate had basically stabilized at a lower level. This result indicates that improving SDI level, especially in socioeconomically underdeveloped regions, may help significantly reduce the health loss caused by NHL.

The DEA frontier line reflects the theoretically achievable lowest DALY rate at a given SDI level, providing an important reference for evaluating the efficiency of disease prevention and control in each country. For the inefficient points above the DEA frontier line, their potential for improvement in controlling the disease burden of NHL can be found through horizontal comparison. Taking the 2021 data as an example (**Figure 5B**), the actual DALY rates of some countries in the coordinate graph are higher than the lowest DALY rates shown by the frontier line at the same SDI level, indicating that these countries still have room for reduction in the disease burden of NHL under the current socioeconomic conditions. Among them, countries such as Malawi, Uganda, South Sudan, and Niue have significantly higher DALY rates than the frontier line. Measures including early diagnosis and improving healthcare accessibility are needed to curb rising inequality in these low-income countries.

### Health inequality analysis and prevalence rate prediction of NHL

**Figure 6:**
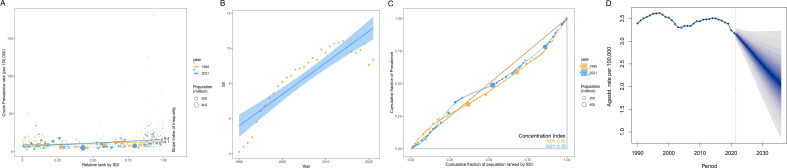
Health inequality analysis and prediction of age-standardized prevalence rates of NHL in children and adolescents. **(A)** Trend chart of changes in the slope index of age-standardized prevalence rates of NHL in children and adolescents from 1990 to 2021. The slope index measures the absolute inequality of the burden of NHL in children and adolescents, and the concentration index measures the relative inequality. The larger the absolute value of the index, the higher the degree of inequality. **(B)** Scatter plot of the slope index of age-standardized prevalence rates of NHL in children and adolescents from 1990 to 2021. **(C)** Inequality curve of age-standardized prevalence rates of NHL in children and adolescents. The horizontal axis represents the cumulative share of the population, and the vertical axis represents the cumulative share of prevalence rates. The dashed line is the reference line. When there is complete equality, the share of prevalence rates for each population is equal to the share of the population. The farther the curve deviates from the reference line, the greater the degree of inequality. **(D)** Prediction of global age-standardized prevalence rate of NHL in children and adolescents from 2022 to 2036.

The results of the health inequality analysis showed that the SII of NHL was 4.1 in 1990 and increased to 9.4 in 2021 (**Figure 6A**). The increase in the absolute value of SII indicates an exacerbation of the absolute inequality of NHL between high and low SDI regions. This means that the burden of NHL has become more concentrated in low SDI regions, while high SDI regions have a relatively lighter burden.

From the SII scatter plot, it can be seen that the slope index of the global ASPR of NHL from 1990 to 2021 shows an overall increasing trend year by year, and the fitted regression line indicates that the long-term trend of the slope index is tilted upward, i.e., the degree of absolute inequality is intensifying (**Figure 6B**).

The CI increased from 0.09 in 1990 to 0.15 in 2021 (**Figure 6C**), indicating that the degree of inequality in the distribution of health resources and disease burden is intensifying. The increase in CI is close to twofold, indicating that health inequalities have significantly worsened over the past 30 years. Both the CI in 1990 and 2021 were positive values, indicating that the prevalence of NHL were mainly concentrated in high SDI regions. The CI was closer to 0 in 1990, indicating that the distribution of prevalence among different SDI regions was more balanced in 1990. The increase in CI further corroborates the trend of exacerbation of relative inequality in NHL.

In summary, both SII and CI showed a clear upward trend, indicating that the distribution of prevalence rates of NHL among different SDI regions is becoming increasingly unbalanced, and health inequalities are becoming more prominent. In addition, the disease burden is mainly concentrated in high SDI regions, and its distribution inequality has significantly worsened compared to 1990.

The BAPC model predicted that the global ASPR of NHL will continue to decrease in the next 15 years (**Figure 6D**). Specifically, the predicted prevalence rate will decrease from 3.177/100,000 in 2021 to 2.048/100,000 in 2036, a decrease of 35.56%. This result indicates that on the basis of existing disease prevention and control measures and medical technology levels, the global burden of NHL is expected to be further controlled and reduced. It should be noted that although the prediction results show a downward trend in future prevalence rates, NHL remains an important disease that threatens the health of children and adolescents.

### Regional distribution of NHL disease burden

**Figure 7:**
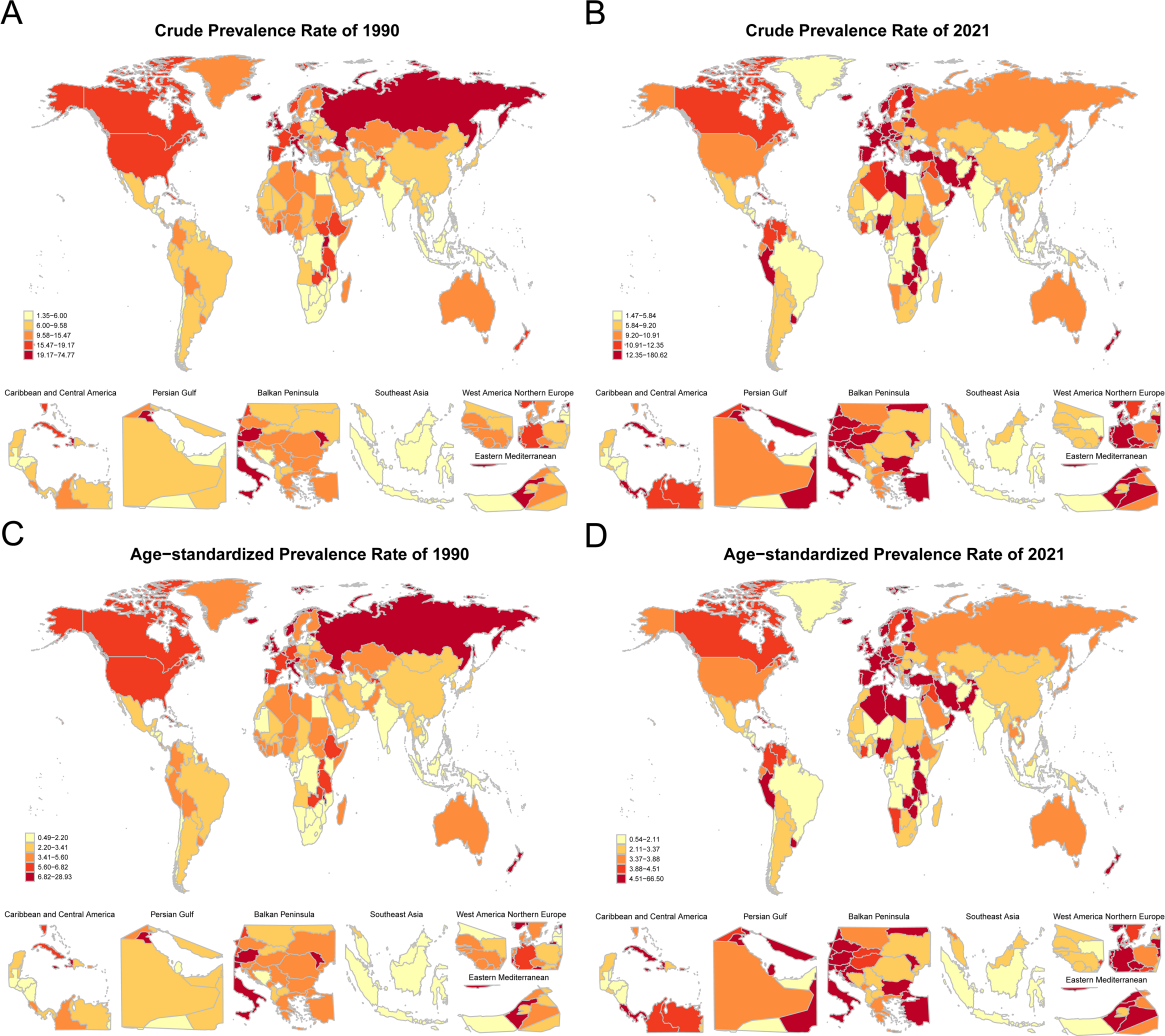
Global distribution of crude prevalence rates and age-standardized prevalence rates of NHL in children and adolescents in 1990 and 2021. **(A)** Distribution map of crude prevalence rates of NHL in children and adolescents in various countries in 1990. **(B)** Distribution map of crude prevalence rates of NHL in children and adolescents in various countries in 2021. **(C)** Distribution map of age-standardized prevalence rates of NHL in children and adolescents in various countries in 1990. **(D)** Distribution map of age-standardized prevalence rates of NHL in children and adolescents in various countries in 2021. Dark-colored areas represent countries with higher age-standardized prevalence rates, while light-colored areas represent countries with lower age-standardized prevalence rates.

Comparing the global distribution maps of crude prevalence rates of NHL between 1990 and 2021, it can be found that the regional distribution pattern of NHL disease burden in children and adolescents has changed to some extent over the 30 years. In 1990, the crude prevalence rates of NHL were higher in regions such as the Republic of San Marino, Republic of Estonia, and Principality of Andorra (**Figure 7A**), while the crude prevalence rates were lower in regions such as the Republic of Mauritius, Republic of Kiribati, and Republic of Cabo Verde. In terms of the trend of ASPRs, the ASPRs of NHL in countries such as the Republic of San Marino, Republic of Estonia, and Principality of Andorra remained high in 1990 (**Figure 7C**), while the ASPRs in island countries such as the Republic of Mauritius, Republic of Kiribati, and Republic of Cabo Verde were at a lower level worldwide.

In 2021, in countries such as the Republic of Mozambique, Republic of Honduras, and Republic of Kiribati, which are mostly located in underdeveloped regions of Africa, Central America, or the central Pacific, the number of patients reported with NHL was relatively small (**Figure 7B**). From the global distribution map of ASPRs in 2021, the ASPRs in low-income or lower-middle-income countries such as the Republic of Mozambique, Republic of Honduras, and Republic of Kiribati were relatively low (**Figure 7D**).

Comprehensively comparing the results of 1990 and 2021, it can be found that the regional distribution pattern of NHL disease burden in children and adolescents has changed to some extent over the 30 years. Overall, the prevalence rates in some African, Central American, and Pacific island countries were relatively low. It is worth noting that the lower prevalence of NHL in less developed countries could be partially attributed to under-reporting or misclassification due to the limitations of healthcare infrastructure, diagnostic facilities, treatment capabilities, and reporting systems in these regions (13).

**Figure 8:**
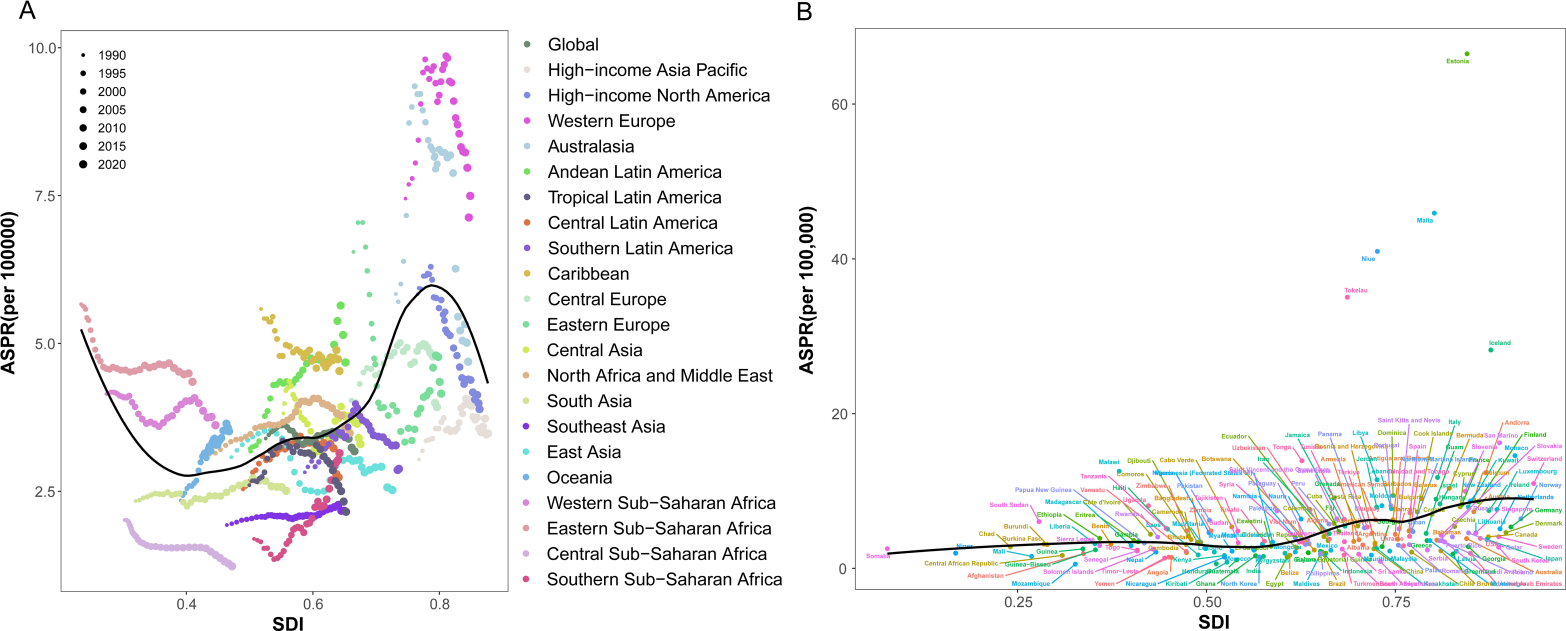
Trends in age-standardized prevalence rates of NHL in children and adolescents in different GBD regions and SDI regions from 1990 to 2021. **(A)** Scatter plot of the relationship between changes in prevalence rates of NHL in children and adolescents and SDI in 21 GBD regions. **(B)** Trend chart of changes in prevalence rates of NHL in children and adolescents in different SDI regions.

To further explore the relationship between SDI and the disease burden of NHL, this study conducted a correlation analysis of SDI and ASPRs in different regions and countries (**Figures 8A, B**). From a regional perspective, the ASPRs of NHL exhibited distinct wave-like patterns as SDI levels improved, characterized by an initial decline, followed by an increase, and subsequently another decrease (**Figure 8A**). This nonlinear relationship suggests that there is a complex interaction between SDI and the prevalence risk of NHL, which may be influenced by multiple factors. From a national perspective, the ASPRs of NHL in middle SDI, high-middle SDI, and high SDI countries were generally higher than the global average level and showed a continuous upward trend (**Figure 8B**). This result indicates that developed regions still face great challenges in the prevention and control of NHL.

## Discussion

Based on the GBD database, this study utilized comprehensive epidemiological and statistical methods to analyze the global burden of NHL among children and adolescents from 1990 to 2021. The analysis revealed that the global age-standardized prevalence showed an overall decline, though with temporal variations. Significant geographic disparities existed, with higher prevalence in European countries and lower rates in African and Central American regions. Age-specific patterns were identified, with population growth being the primary driver of prevalence increases. These findings provide valuable insights for developing targeted NHL prevention and control strategies globally.

The results of this study showed that the global disease burden of NHL in children and adolescents generally presented a fluctuating downward trend from 1990 to 2021. Interestingly, a previous study based on the GBD database analysis found that from 1990-2017 the incidence of NHL globally was increasing (11). But that study focused on the data of NHL in the population over 15 years old which is not suitable to reflect the changing trends among children and adolescents. Since the end of 2019, the COVID-19 pandemic has caused multiple lockdowns and widespread restrictions, which directly or indirectly increased the delay in the diagnosis of childhood cancer (14). Some study observed a 28% reduction for lymphoma diagnoses overall during the COVID-19 pandemic period (15), while other indicated that the incidence of lymphoma remained unchanged (16). No increasing in incidence of pediatric NHL was found during the pandemic. Therefore, it cannot be denied that from 2019 to 2021, the COVID-19 pandemic to some extent led to a decrease in the number of diagnoses of childhood NHL, thereby reducing the number of registrations. Eventually, the statistics show that the prevalence rate showed a downward trend during this period.

In the early 21st century, there was a brief rebound in the global prevalence of NHL in children and adolescents, which may be related to improvements in medical conditions and diagnostic levels, such as the application of positron emission tomography-CT and magnetic resonance imaging technology, which helps early detection of lymphoma (17). In addition, disease monitoring and reporting systems in various countries are constantly improving, making prevalence data more accurate and comprehensive (18). Moreover, in this study, the prevalence of NHL in male children was consistently higher than that in females, which is consistent with previous reports (19). That study showed that the overall prevalence of NHL in females was about 30% lower than in males (19). This may be related to inherent differences in susceptibility between genders, and other factors may also play a role as age increases. Previous studies have shown that males have a higher risk of most cancer types compared to females (20). In some cancers, such as lung cancer and head and neck cancer, this difference is partly due to higher smoking and alcohol consumption rates among males (21). In other cancers, including hematological malignancies, the underlying mechanisms remain unclear (21). Helicobacter pylori, Epstein-Barr virus and Hepatitis C infections have been closely associated with the development of non-Hodgkin lymphomas, and the infection rate is higher in males than in females (5, 22). Some studies suggest that gender differences in immune surveillance may make males more susceptible to proto-oncogene mutations and more vulnerable to chronic, potentially carcinogenic infections (23). There is also evidence that the number of pregnancies is negatively correlated with the risk of NHL, suggesting that sex hormones may influence the occurrence of lymphoma (24). **Figure 1** shows that in recent years, the rate of decline in the prevalence of NHL in children and adolescents has slowed down. This reflects that the incidence of NHL has increased gradually, which may be related to changes in lifestyle, infections or transplantation affecting immune function, and increased environmental pollution (13, 22, 25–27). This may also indicate that existing prevention and control measures have reached a certain bottleneck (17, 28, 29). Meanwhile, with the improvement of medical conditions in low-income areas, more children with NHL may have been diagnosed in time.

This study found that age had a significant impact on the risk of NHL in children and adolescents, with the risk declining with age before 12.5 years and rebounding thereafter. This result suggests that young children and adolescents are high-risk age groups for NHL. The reasons for this trend may be that the immune system gradually matures during development, enhancing the ability to resist viral infections and abnormal cell proliferation; exposure to certain infections (such as EBV) decreases with age; certain genetic susceptibilities are more significant in childhood and their influence may weaken with age; and with age, cell repair mechanisms become more mature and stable, thus reducing the risk of NHL (30–32). The slight rebound in risk after adolescence may be related to factors such as vigorous secretion of sex hormones and increased chances of HIV infection after puberty (33). It is worth mentioning that B-cell NHL is considered an AIDS-defining malignancy, and related studies have shown that regions with high prevalence of lymphoma also have high prevalence of HIV (34). The above results suggest that prevention and control of NHL in children and adolescents should focus on carrying out targeted health education and disease screening. In terms of period effects, improvements in medical conditions and recent progress in the treatment of NHL may improve overall outcomes instead of decrease the prevalence (29).The rebound trend since 2004 is noteworthy, indicating that the adverse effects of modern lifestyles and environmental issues on NHL in children and adolescents cannot be ignored. At the same time, improvements in medical conditions, standardization of diagnostic procedures, and advancements in diagnostic technologies such as imaging may also improve the detection rate of NHL to some extent (35). Even if the increase in the prevalence of NHL in children and adolescents is inevitable, to substantially improve survival, it is necessary to put investments to expand access to multidisciplinary care, to improve healthcare quality and improve service delivery (36). Our cohort effect analysis results showed significant differences in the risk of NHL in children and adolescents between different birth cohorts. Among them, the risk was relatively high for birth cohorts in the 1970s and 1980s, while the risk gradually decreased for birth cohorts after the 1990s. Changes in environmental factors between birth cohorts may largely influence the prevalence of NHL. For example, children and adolescents born in the 1970s and 1980s may have been exposed to higher levels of environmental carcinogens during their growth, such as agricultural chemicals, radiation, pesticides, and the prevalence of certain viral infections (such as EBV), which have been proven to be closely related to the occurrence of NHL (37, 38). Since the 1990s, with the strengthening of environmental governance and the improvement of public health interventions, exposure to related carcinogenic factors has decreased, which may lead to a reduction in the risk of disease.

The results of this study showed that population growth was the primary driver of the increase in the prevalence of NHL in children and adolescents. Population growth directly leads to an increase in the absolute number of children and adolescents, thus increasing the potential number of NHL cases. Population aging has a complex impact on prevalence changes by influencing the per capita risk of disease. On the one hand, as life expectancy increases and fertility rates decrease, the proportion of children and adolescents in the population decreases, and the prevalence of NHL may also decrease accordingly. On the other hand, in the context of population aging, with the rapid development of the socioeconomic system, changes in lifestyle, increased environmental pollution, and increased prevalence of chronic diseases may offset some of the positive effects of population aging (39, 40). Improvements in medical conditions, optimization of diagnosis and treatment plans, and other epidemiological factors (41, 42) may help reduce the risk and mortality of NHL in children and adolescents. These complex mechanisms of influence suggest that when formulating prevention and control strategies for NHL in children and adolescents, we should not only pay attention to the objective impact of changes in population size and structure but also fully consider the dynamic changes in lifestyle, environmental factors, and disease spectrum in the process of socioeconomic development, and then take targeted comprehensive prevention and control measures.

The DEA analysis results of this study showed that the DALY rate caused by NHL in children and adolescents had a nonlinear negative correlation with SDI, and as SDI increased, the DALY rate showed an overall downward trend, indicating that improving socioeconomic development levels and strengthening medical security may help reduce the health loss caused by NHL in children and adolescents. Data show that cancer causes more than 100,000 child deaths each year, with over 90% occurring in low- and middle-income countries (43). These countries often have scarce medical resources and weak health systems, and access to education, modern technology, and health services is limited due to the dispersal of the population in rural areas (44). At the same time, treatment abandonment is the main reason for treatment failure in children with cancer in these countries (45). In contrast, in high-income countries, about 80% of children with cancer can be cured through timely, intensive multimodal treatment and strong supportive care (44). The slowing rate of decline in DALY rates as SDI increases suggests that although socioeconomic development and improved medical conditions help reduce the health loss caused by NHL in children and adolescents, this positive effect may be offset by new risk factors such as modern lifestyles and increased environmental pollution once SDI reaches a high level.

The results of the health inequality analysis showed that both the absolute and relative inequality in the prevalence of NHL in children and adolescents globally increased from 1990 to 2021, with higher prevalence in high SDI regions and lower prevalence in low and middle SDI regions. This is consistent with previous research results, which indicated that the regional distribution of NHL showed significant differences, with prevalence increasing in countries with high human development index (HDI) and high per capita GDP (13). These regions usually have higher rates of sedentary lifestyles and obesity (46, 47), which are known risk factors for NHL (48, 49). The widening health inequality stems from the interplay of multiple factors. From a medical technology perspective, more advanced medical diagnostic technologies and screening systems in high SDI regions significantly improve the detection rate of NHL, while limitations in this regard in low and middle SDI regions may result in underdiagnosis or misdiagnosis (50). Moreover, disparities in healthcare accessibility, such as limited availability of pediatric oncology specialists, diagnostic equipment, and timely medical services, further compound the inequality. In many low- and middle-SDI regions, inadequate healthcare infrastructure, workforce shortages, and financial barriers delay diagnosis and limit treatment options. From a biological and socioeconomic perspective, genetic studies have confirmed significant differences in genetic susceptibility among populations in different regions, and the interaction of these biological factors with socioeconomic conditions further exacerbates health inequalities (51). In addition, recent diagnostic advancements, including flow cytometry, immunophenotyping, and molecular profiling, have improved early detection and subtype classification in high-income settings, but these tools remain largely inaccessible in resource-constrained areas. Overall, health inequality is not only a challenge in the medical field but also a complex systemic social problem closely related to education, economic development, social infrastructure, and other multiple factors (44). Therefore, to reduce the prevalence gap of NHL in children and adolescents across regions, it is essential to implement holistic and cross-sectoral intervention strategies.

This study predicted that the global age-standardized prevalence of NHL in children and adolescents would further decline from 2022 to 2036. This optimistic estimate indicates that existing preventive healthcare measures, screening and diagnostic technologies, and standardized treatment regimens will continue to play a positive role in the future period. For example, the widespread application of innovative therapies such as immunotherapy worldwide (41, 42) would improve the survival of NHL in children and adolescents, and the optimization of early screening and prevention strategies for high-risk groups of NHL (52) are expected to increase early detection of subclinical lesions of NHL or reduce the risk of NHL in children and adolescents. However, this overall downward trend needs to be viewed dialectically, and the combined effects of the potential impact of modernized lifestyle changes (46, 47, 53) and environmental pollution (54–56) may affect the epidemic trend of NHL in children and adolescents to some extent. Though how to maintain and accelerate this downward trend will be an important topic in the global prevention and control of NHL in children and adolescents, early diagnosis and more diagnosis in low-income countries is probably key to improving their outcomes.

This study has several limitations. There are potential limitations of the GBD data for pediatric NHL. First, the accuracy of the GBD estimate largely rely on the availability and quality of the data. There’s potential variability and inconsistency of cancer registry data across countries which might influence temporal trends of childhood cancer. Data on certain childhood cancers, including NHL, from some regions or countries might be sparse or even absent in the GBD dataset (57). High-quality data in many regions and countries were sparse, particularly in low-income locations (58). Our study lacks complete data on children under the age of 5, with only data on children aged 12 to 23 months. The statistical data on NHL in children and adolescents are missing for some countries and regions in the GBD database, which may affect the accuracy and representativeness of the results. In the future, the collection and management of monitoring data on NHL in children and adolescents should be further strengthened, the information reporting mechanism should be improved, and data quality should be enhanced. Second, in years where data were not available, estimates of GBD relied on covariates and modeling parameters may overestimate or underestimate true cancer burden (59). The prediction model used in this study is mainly based on extrapolation of historical epidemiological data, and there may be bias in the judgment of the future situation of prevention and control of NHL in children and adolescents. In the future, various prediction methods should be comprehensively considered, prediction plans should be dynamically adjusted, and the scientific nature and accuracy of predictions should be continuously improved. Third, this study mainly explored the impact factors at the macro level, such as population demographics, socioeconomics, and time effects, and the analysis of micro-level impact factors such as genetic susceptibility, immune function, and viral infections is not in-depth enough. In the future, more comprehensive and systematic etiological studies should be conducted on this basis.

## Conclusion

This study revealed a fluctuating downward trend in global NHL burden among children and adolescents from 1990 to 2021, with persistent regional disparities and increasing health inequalities. Though there’s socioeconomic disparities between the high-income countries and the low-income countries, better healthcare accessibility and diagnostic advancements can benefit the children and adolescents of NHL worldwide with better outcomes. Future efforts should focus on targeted prevention strategies, enhanced management of high-risk populations, and improved disease screening programs. Strengthening etiological research and risk factor intervention remains crucial for reducing NHL burden and promoting health equity among children and adolescents globally.

## Data Availability

The original contributions presented in the study are included in the article/supplementary material. Further inquiries can be directed to the corresponding author.
